# Intricate Correlation between Body Posture, Personality Trait and Incidence of Body Pain: A Cross-Referential Study Report

**DOI:** 10.1371/journal.pone.0037450

**Published:** 2012-05-18

**Authors:** Sylvain Guimond, Wael Massrieh

**Affiliations:** 1 San Diego University for Integrative Studies, San Diego, California, United States of America; 2 Division of Experimental Medicine, Department of Medicine, McGill University, Montreal, Québec, Canada; Catholic University of Sacred Heart of Rome, Italy

## Abstract

**Objective:**

Occupational back pain is a disorder that commonly affects the working population, resulting in disability, health-care utilization, and a heavy socioeconomic burden. Although the etiology of occupational pain remains largely unsolved, anecdotal evidence exists for the contribution of personality and posture to long-term pain management, pointing to a direct contribution of the mind-body axis. In the current study, we have conducted an extensive evaluation into the relationships between posture and personality.

**Method:**

We have sampled a random population of 100 subjects (50 men and 50 women) in the age range of 13–82 years based on their personality and biomechanical profiles. All subjects were French-Canadian, living in Canada between the Québec and Sorel-Tracy areas. The Biotonix analyses and report were used on the subjects being tested in order to distinguish postural deviations. Personality was determined by using the Myers-Briggs Type Indicator questionnaire.

**Results:**

We establish a correlation between ideal and kyphosis-lordosis postures and extraverted personalities. Conversely, our studies establish a correlative relationship between flat back and sway-back postures with introverted personalities.

**Conclusion:**

Overall, our studies establish a novel correlative relationship between personality, posture and pain.

## Introduction

Complications with posture and back pain are projected to become a widespread medical and socio-economic issue across the globe, with more than 70% of the population predicted to be engrossed in the problem [1]. Acute back pain affects every year up to 45% of the population between the ages of 35 and 55 years [2], with 2 to 7% of this cohort exacerbating to chronic back pain [3]. It has been estimated that 1 in 25 people will change his or her work because of low back pain or will retire early due to disability stemming from low back pain [4]. Back pain has been found to be the most expensive non-cancerous condition in industrialized countries, while representing the primary cause of disability under the age of 45 years [4], [5]. In the United States, 6–8% of the adult population has been found to have back pain at any given time. The prevalence rises after age 25 to peak in the 55–64-year range, with the rate falling after age 65 [5]. The major shortcomings in preventing and treating back pain are inadequate treatment regimens and more importantly lack of preventable measures. Hence, it is imperative to properly understand the physical aspects that contribute to pain and misalignment along with the behaviors contributing to such misalignment in order to be able to appropriately modify behaviors in order to prevent and/or cure back pain.

Posture plays a significant role in back pain and refers to our dynamic, adjustable, and responsive positioning to the environment [6], [7]. Each body segment has a center of mass, the different segments forming a composite center of mass that, in turn, creates a center of gravity [8], which helps in maintaining body balance with minimal effort. However, misalignment of certain body segments as a result of postural deviation will cause compensatory effort by other segments to maintain body balance, resulting in muscular strains and stress on the neurological system and resulting in back pain [9], [10]. According to Kendall and Kendall, there are four major types of posture. The first posture is ideal posture, the second is kyphosis-lordosis, the third is flat back, and the fourth is sway-back [11]. It seems that the body shapes itself into different postures depending on the underlying mental and emotional state, thus establishing a direct link of the body-mind axis and posture [12].

The mental and emotional state can be collectively termed the personality, a stable set of characteristics that appear in individuals in unique combinations and account for their cognitions, motivations, and behaviors [13]. Personality type refers to the psychological classification of different types of people according to their preferences, tendencies, and behavioral consistencies [14]. Based on Jung's Theory of Personality Preferences, Katharine Briggs and Isabel Briggs Myers designed the Myers-Briggs Type Indicator (MBTI), a psychological tool to scientifically assess 16 different personality types [15], [16].

MBTI contains four separate dichotomies: Extraversion-Introversion, Sensing-Intuition, Thinking-Feeling, and Judging- Perceiving [16]. Sensing-Intuition and Thinking-Feeling describe mental functions and reflect basic preferences for use of perception and judgment, whereas Extraversion-Introversion and Judging- Perceiving reflect attitudes or orientations [16]. Together, these functions and orientations influence how a person perceives a situation and decides on a course of action.

Previous research has shown that happy thoughts lead to more upright postures, while sad thoughts lead to slumped and hunched positions [16]. In turn, upright, posture can alleviate depression by improving breathing. This lead to increased oxygen levels in the blood and subsequently relieves muscular tension in the shoulders [17]. However, the exact mechanism of how posture affects our moods is not well understood. Hence, the purpose of the current study was to identify whether a relationship exists between posture and personality and in turn whether it has predictive value for back pain. The BioPrint system was used to predict posture of subjects based on Kendall and Kendall's classification and the data was integrated to the findings of the Myers-Briggs analyses. Our studies establish a direct relationship between posture and personality and a correlative relationship between personality, posture and pain. Importantly, our data significantly points to a strong interplay of conscious sensation and involuntary actions, reiterating the prevalence of the mind-body axis.

## Results

### Subject Analysis

**Figure 1:**
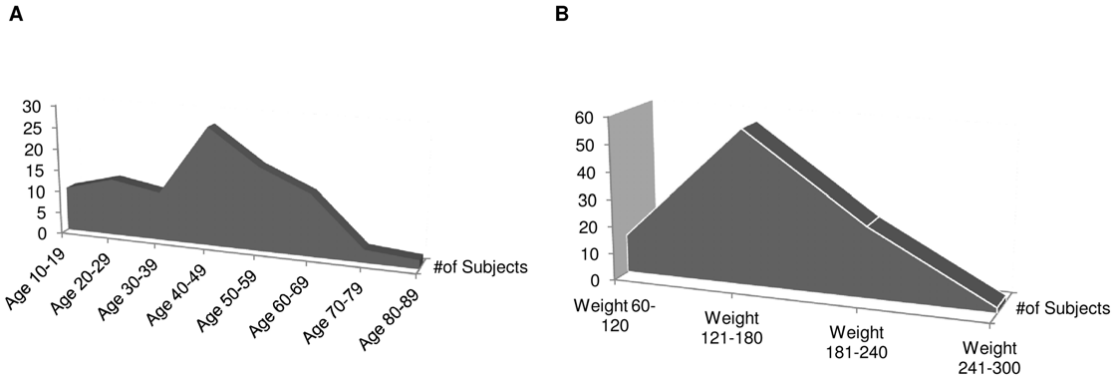
Distribution of age and weight classification of the study group. The graphs depict a non-biased age (*A*) and weight distribution (*B*) for the subjects evaluated for the study.

One hundred persons were randomly selected. The selected subjects had a mean age, height, weight, and BMI of 44.17±16.67 years, 65.81±3.8 inches, 160.18±36 pounds and 25.86±4.91, respectively. The distribution of the age and weight of the test population, represented in **Fig. 1A** and **B**, respectively, depicts a random distribution without specific partialization to any age or weight groups. Results were collected from all three areas of assessments upon completion of 100 subjects. The results from the personality inventory (MBTI) came in the form of a combination of letters [Extraversion, Introversion, Sensing, Intuition, Thinking, Feeling, Judging, and Perceiving], and numbers, 1–26, both in raw scores and in the final analysis (**Table S1**). The postural evaluation results came in the form of numbers (**Table S1**). The numbers represented data taken from the BioPrint photographs, measuring angles related to each subject's posture. The pain scale data consisted of a number between 0 and 10 (**Table S1**). After the compilation of all the results in the three categories of testing, one of the four letters [ideal posture (A), kyphosis-lordosis (B), flat back (C), sway-back (D)] was added to the Myers-Briggs Personality Type Indicator (MBTI) test results. The purpose of adding the letter was to create a new formula for the purpose of the current study, comparing personality type with posture type. Twenty-two of the subjects grouped in ideal posture, 36 in kyphosis-lordosis posture, 19 in flat back posture, and 23 in sway-back posture.

### Personality distribution of the tested subjects

**Table 1** summarizes the 16 personality types according to the MBTI Manual [16] and represents the posture group [ideal posture, kyphosis-lordosis, flat back, and sway-back] into which each personality type falls. Except for the INTP (Introversion- Intuition-Thinking-Perceiving) type, subjects in this research represented all of the MBTI personality types. Overall, 65% of the subjects tested as extraverted and 35% as introverted.

**Table 1:** Distribution of the 16 different personality types, based on the MBTI Manual, and the observed number of subjects from each posture group (A, B, C, or D) that falls under each personality type.

MBTI Type	A	B	C	D	Total
***ESTJ***	2	3	0	2	7
***ESTP***	3	6	1	0	10
***ESFJ***	1	6	2	2	11
***ESFP***	5	5	2	0	12
***ENTJ***	0	2	1	2	5
***ENTP***	1	3	1	0	5
***ENFJ***	1	3	1	0	5
***ENFP***	8	2	0	0	10
***ISTJ***	0	2	1	5	8
***ISTP***	0	2	1	1	4
***ISFJ***	1	2	3	0	6
***ISFP***	0	0	2	6	8
***INTJ***	0	0	1	0	1
***INTP***	0	0	0	0	0
***INFJ***	0	0	1	2	3
***INFP***	0	0	2	3	5
**Total**	**22**	**36**	**19**	**23**	**100**

Posture A – Ideal; Posture B – Kyphosis-Lordosis; Posture C – Flat Back; Posture D – Sway-back.

The aim of the MBTI assessment was to clarify personality type theory and make it accessible to individuals and groups. The MBTI consisted of the following four dichotomies (I–IV) [15]: [I] *Favorite world*: preference to focus on the outer world or on own's inner world, called Extraverted or Introverted, respectively; [II] *Information*: preference to focus on the basic information available or preference to interpret and add meaning, called Sensing or Intuition, respectively; [III] *Decisions*: preference to first look at logic and consistency or first look at the people and special circumstances, called Thinking or Feeling, respectively; [IV] *Structure*: preference to get things decided or stay open to new information and options while dealing with the outside world, called Judging or Perceiving, respectively.

Subsequently, we evaluated combinations of MBTI preferences compared with the different posture categories. The MBTI had 93 questions with two possible preferences for each question. For example, to determine an Extraverted preference or an Introverted preference, there were 21 questions throughout the questionnaire in that category. If the subjects choose all of those 21 questions as Extraverted preference, the score would be twenty-one for Extraverted preference and zero for Introverted preference. If the subject chose seven questions in Introverted preference the score would be fourteen for Extraverted and seven for Introverted. By summing the total score for each preference category, the average score of type preference for that subject was determined. The MBTI preferences were a significant factor in how the subject related to his or her environment. The four mental functions were called temperaments. The Sensing-Judging, Sensing-Perceiving, Intuition-Feeling, and Intuition-Thinking do not influence posture or orientations; they influence decision and not orientations. As summarized in **Table 2**, our data analyses not only establish a correlative relationship between posture and personality, but also demonstrate each personality function has significant influence on the individual's posture.

**Table 2:** The average score of preference for each of the one hundred subjects tested.

Preference	A	B	C	D
***E***	17	14	11	8
***I***	4	7	10	13
***S***	15	15	15	16
***N***	11	11	11	10
***T***	9	11	10	12
***F***	15	13	14	12
***J***	7	11	12	13
***P***	15	11	10	9

It is the score for each of the eight possible preferences and the posture category the subjects are classified into.

**Figure 2:**
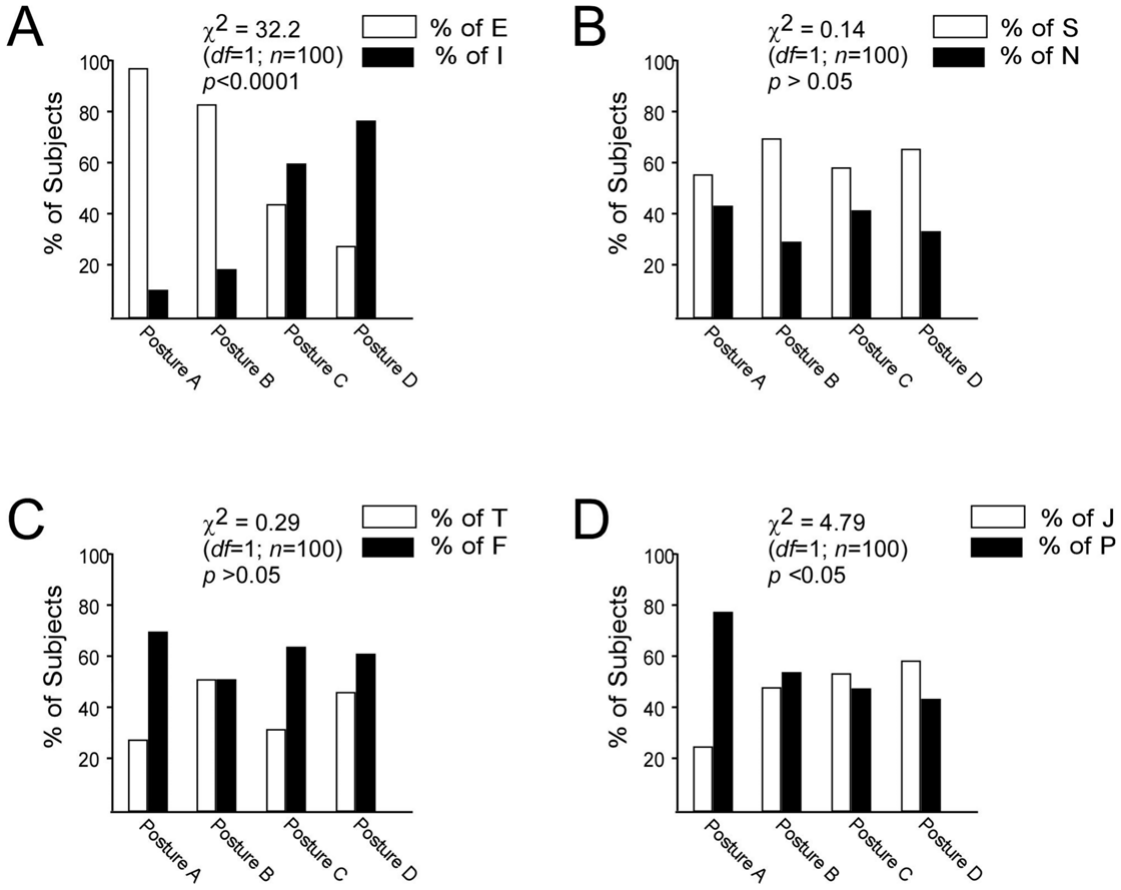
Correlation of personality types and the four different posture types. In ideal posture (A), there were 96% of Extraverted subjects, kyphosis-lordosis posture (B) had 83% of Extraverted preference, flat back posture (C) had 42% and sway-back posture (D) had 26% (*A*). Percentage of Sensing and Intuitive Preferences did not differ by posture category (*B*). There was no significant relationship between Thinking (T) and Feeling (F) preferences, and posture (*C*). However, Judging (J) or Perceiving (P) preferences significantly varied with the posture types (*D*).

As shown in **Fig. 2A**, the proportion of Extraverted and Introverted preference varied considerably among the four posture groups. These differences were significant (χ^2^ = 32.2; *df* = 1; *n* = 100; *p*<0.0001), thus giving general support to the overall hypothesis that posture differences are related to personality variables. This demonstrated the direct relationship between Extraverted and Introverted subjects with the four different posture categories represented in this research. In ideal posture, 21 of the 22 subjects were Extraverted, meaning that 96% of ideal posture subjects were Extraverted. Only one person in ideal posture was Introverted, meaning that only 4% of ideal posture subjects were Introverted. In kyphosis-lordosis posture, 30 subjects were Extraverted and only 6 were Introverted, making 83% of kyphosis-lordosis posture subjects Extraverted and 17% Introverted. In flat back posture, only 8 subjects were Extraverted and 11 were Introverted, resulting in 42% Extraverted and 58% Introverted. Finally, in sway-back posture, 6 subjects were Extraverted and 17 were Introverted, meaning that 26% of sway-back posture were Extraverted and 74% were Introverted. In summary, our results clearly demonstrate a relationship between a person's demeanor and their posture.

Of note, Sensing and Intuition preference did not vary considerably among the four posture groups (**Fig. 2B**). The observed differences were not significant (χ^2^ = 0.14; *df* = 1; *n* = 100; *p*>0.05), thus giving general support to the overall hypothesis that posture differences are not related to these more internal personality variables. Our data also showed the representation between Sensing and Intuition preference in relation to the four postures tested. Chi-square test did not reveal any relationship between posture and the perceiving functions of Sensing and Intuition (**Fig. 2B**). No significant relationship (χ^2^ = 0.29; *df* = 1; *n* = 100; *p*>0.05) between Thinking and Feeling preferences and posture were observed (**Fig. 2C**). Extraverted types were best represented in ideal posture, whereas Introverted types best represented in sway-back posture, which represents the posture with the most deviation. Overall, our data demonstrated that posture and personality types are highly correlated in the Extraverted/Introverted dimension, but not in the Thinking/Feeling dimension. According to Kendall [11], people with ideal posture have less tension and contraction than other postures types. Because of their character, people with a Feeling preference are more relaxed and easy-going than those who exhibit Thinking preference. Their characters affect their posture and vice versa and our findings corroborate the same

Proportion of subjects who utilized Judging or Perceiving preferences also varied considerably among the four posture groups (**Fig. 2D**). The differences were significant (*χ^2^* = 4.79; *df* = 1; n = 100; *p*<0.05) and, thus, give general support to the overall hypothesis that posture differences are related to personality variables. The majority (77%) of ideal posture subjects leaned towards Perceiving preference, whereas it was more uniform for kyphosis-lordosis posture (47% Judging and 53% Perceiving), flat back posture (53% Judging and 47% Perceiving), and sway-back posture (57% Judging and 43% Perceiving).

**Figure 3:**
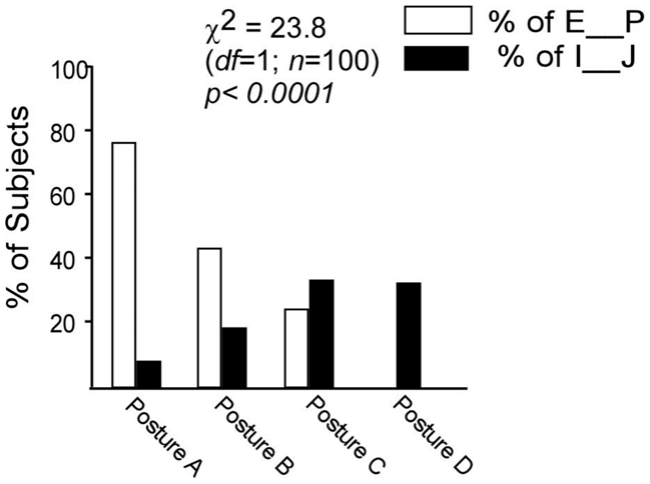
Graph of Adaptable Extraverts and Decisive Introverts. Further analysis indicated that there is a relationship between posture and the Adaptable Extraverts: ESTP, ESFP, ENFP, ENTP (E_ _P) as well as the Decisive Introverts: ISTJ, ISFJ, INFJ, INTJ (I_ _J) (*Extraversion-Introversion (E-I), Sensing-Intuition (S-N), Thinking-Feeling (T-F), and Judging- Perceiving (J-P)* [*16*] *. Sensing-Intuition (S-N) and Thinking-Feeling (T-F) describe mental functions and reflect basic preferences for use of perception and judgment, whereas Extraversion-Introversion (E-I) and Judging- Perceiving (J-P) reflect attitudes or orientations*).

The proportion of Extraverted Perceivers versus Introverted Judgers varied with the four posture groups (**Fig. 3**). The differences in results were significant (*χ^2^* = 23.8; *df* = 1, *n* = 100; p<0.0001), thus giving general support to the overall hypothesis that posture differences are related to personality variables. Extraverted Perceiving preference, the Adaptable Extraverted, and the Introverted Judging preference, which use perception to deal with the outer world, also showed a relationship between personality type and posture type. The percentage of Extraverted versus Introverted in each posture established a correlative relationship between posture and personality. Additionally, it also indicates that the cognitive functions may have a limited relationship with posture.

### Relationship between posture and pain

**Figure 4:**
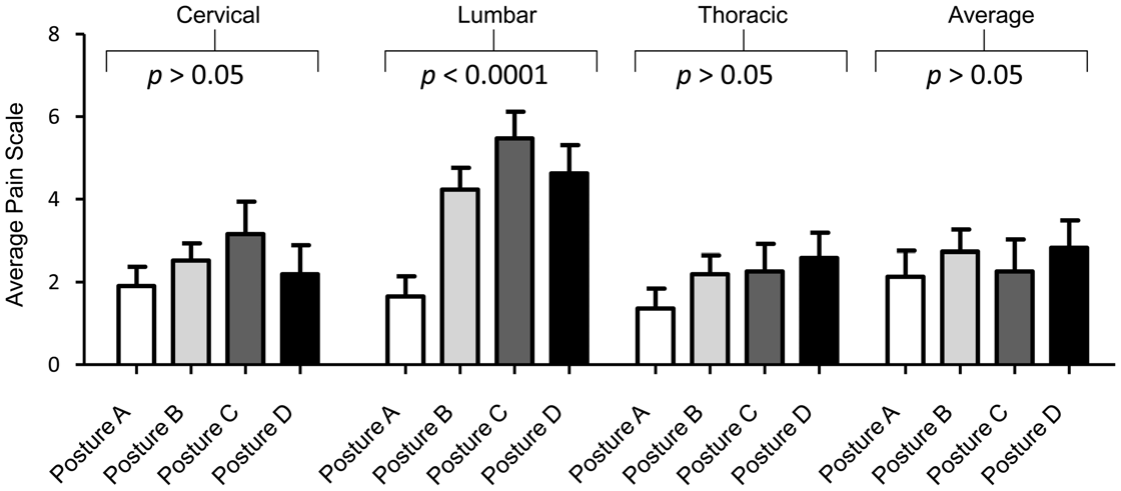
Subjects with ideal posture (A) reported the least amount of pain in the lumbar region. Subjects were asked to rate pain in the cervical, lumbar and thoracic regions on a scale of 0–10, with 10 signifying the most painful condition. There was no significant correlation between cervical and thoracic pain with posture. All other subjects, except for those in ideal posture, experienced more pain in all the observed areas. (*A, Ideal posture; B, Kyphosis-lordosis posture; C, Flat back posture; D, Sway-back posture*).

Each subject was asked to fill out a pain scale questionnaire. The subjects were additionally asked to identify the location of the pain (cervical, thoracic or lumbar), as well as the intensity of the pain, on a scale from zero- no pain, to 10- extremely painful. **Fig. 4** and **Table 3** summarize the average pain felt by each subject in each of the four postures. Subjects in ideal posture had significantly less back pain (lumbar pain) than subjects in other three postures (*p*<0.0001). Similar significant correlation was not observed for cervical and thoracic region pain incidence, even though subjects in ideal posture had overall less pain incidence in all three groups.

**Table 3:** Summary of the relationship between body pain (average of cervical, thoracic and lumbar areas for each type) and posture type.

		CERVICAL	LUMBAR	THORACIC	AVERAGE
	***Mean***	1.909	1.659	1.364	2.136
**Posture A**	***Standard deviation***	2.180	2.265	2.279	2.900
	***Standard Error***	0.4648	0.4828	0.4859	0.6182
	***Mean***	2.528	4.236	2.194	2.736
**Posture B**	***Standard deviation***	2.429	3.150	2.718	3.190
	***Standard Error***	0.4048	0.5250	0.4531	0.5317
	***Mean***	3.158	5.474	2.263	2.263
**Posture C**	***Standard deviation***	3.420	2.836	2.884	3.331
	***Standard Error***	0.7846	0.6505	0.6616	0.7641
	***Mean***	2.196	4.630	2.587	2.826
**Posture D**	***Standard deviation***	3.319	3.290	2.891	3.168
	***Standard Error***	0.6921	0.6860	0.6027	0.6606

Posture A – Ideal; Posture B – Kyphosis-Lordosis; Posture C – Flat Back; Posture D – Sway-back.

## Discussion

The goal of the current study was to investigate the connection between the mind and the body, trying to find a relationship between personality and posture types. Our data uncovers a yet undefined correlative relationship between mind and body, connecting personality type with posture. Since we show a link between the parts of the whole, we can imply that the whole, which in this case is the mind and the body, are connected too.

Our results establish a correlative relationship between each type of posture and the combination of two personality type dimensions. Interestingly, as opposed to common belief that a good posture precludes the body to be straight, tight, and rigid, when actually our results indicate that the opposite is true Personalities that are less flexible and adaptable may correlate with a posture that is less flexible and relaxed. Therefore, a decrease in number of Adaptable Extraverts preferences was present in each of the four different postures considered. According to Kendall and Kendall [11], muscles are most relaxed and less contracted in the ideal posture. Our results corroborate the same. Of note, our results did not reveal any relation between mental processes and posture types.

22% of the subjects tested had a good posture and 19 out of the 22 subjects did not have back pain. Recent studies have investigated how personality types and psychosocial stress influence the functioning of the biomechanical system and subsequent spine loading [18]. Introverts appeared to have one of the largest reactions to psychosocial stress, demonstrating increases in normalized compression and lateral shear [15].

Previous research has demonstrated that people with Type A Personality are more susceptible to heart attacks [19]. Our results have the potential to add an additional dimension of data in the field of biomechanics that will be useful in categorizing each individual patient's susceptibility to back pain. Our work adds to the growing intuition that developing a good personality is truly an attempt to elevate the status of our posture and vice versa [20].

We encompassed MBTI in our study because of its extensive breadth of application. The major disadvantage of MBTI is that it measures the part of the psyche relating to consciousness and cognitive behavior, not motivations, and hence, the results can be wrongly interpreted if not analyzed properly. To rule out bias from our analyses and subsequent interpretations, we also used the Enneagram Type [21] and obtained similar results (data not shown). Of the nine Enneagram types, most were concentrated in one or two MBTI types. Yet another form is the Neuroticism-Extroversion-Openness Inventory (NEO PI-R) with 240 different personality facets [22]. In a comparative study between the NEO PI-R and MBTI system, it was seen that they did not correlate in most facets [21], [22]. Of note, activity levels were not controlled for in our study design and this might be an important variable in determining posture type (e.g. vs. sedentary people). Therefore, future studies, thus, will focus on hierarchical multiple regressions or structural equation modeling that will allow us to perform iterative cycle of system testing and implementation, that will ultimately help us in practical realization of the benefits of the study outcomes. Future endeavors will also study if posture alteration through exercise and awareness also affects personality type. Concurrently, how such remodelling will affect personality in the long term will determine the feasibility of such alternative, yet rational approaches.

## Materials and Methods

### Subjects

100 subjects (50 men and 50 women) in the age range of 13–82 years were randomly selected for the study. All subjects were French-Canadian, living in Canada between the Québec and Sorel-Tracy areas. A protection of human subjects review form in French was completed by all the participants. All subjects were asked to sign a consent form that explained in detail the entire procedure and goal of the study. Names and personal identification of the subjects were duly substituted by a numerical value ranging from 1 to 100. The risks surrounding this study were minimal, considering no invasive measurements were obtained. Data collection was conducted in one day, October 10, 2006, at the Dr. Guimond's Sports Medicine Clinic located in Quebec, Canada. Assessment in three different areas were conducted: personality type (MBTI questionnaire), biomechanical assessment (Biotonix evaluation), and a pain scale questionnaire (provided by the research group). Three certified evaluators from Biotonix were in charge of placing the reflective markers on all of the subjects. A supervisor from Biotonix was in charge of taking all of the pictures for the postural evaluation. Two people were responsible for the MBTI testing of all of the subjects, and were supervised by Dr. Cristina Versari, Ph.D., from the San Diego University for Integrative Studies. The Ethics Committee at the San Diego University for Integrative Studies approved all studies at the Sports Medicine Clinic and the Biotonix Clinic.

### Posture Evaluation

The postural evaluation was carried out in static position by using a digital camera to capture the necessary images. The markers used for the postural evaluation were equipped with a hypoallergenic adhesive, decreasing the possibility of an allergic reaction. Beginning at the marker placement station, the subjects were instructed to wear tight-fitted clothing, such as a swimsuit, for easy application of the markers. Participants completed the relevant questionnaire while seated in a chair at a table in a well-lit room. The Biotonix analyses and report were used on the subjects being tested in order to distinguish postural deviations. Following any identified deviation, each subject was given a 10-week personalized corrective exercise program based on the outcome of his or her BioPrint evaluation. Two weeks later, all subjects who participated in the study also received a copy of their Biotonix evaluation of their posture measurements.

### Variables

Out of the 100 subjects selected, 65% were categorized as extraverted and 35% were introverted. Subjects were asked to appear at a private sports medical clinic to meet new people. An extraverted person was more likely to commit to such an obligation whereas an introverted person would actively avoid it. The predominance of extraverted subjects in the overall study can be explained by the fact that some of the subjects were members of one family, having similar patterns in personality type and physique. Non-risk variables including age, sex, and geographic area of residence were also recorded.

### Criteria and Criteria Measures

Subjects were asked to answer a questionnaire regarding any pain that they were experiencing in their neck, thoracic, lumbar and any other body parts with tension. They were asked to rate their pain based on their personal pain scale from zero to 10, 0 meaning no pain and 10 meaning extreme pain. The first instrument used for this study was the BioPrint System (Biotonix Inc, Montreal, QC, Canada), a biomechanical assessment of posture. The Biotonix's video system had high degree of reliability and validity [23], and, thus, this system was suitable for clinical use in the analysis of posture. The procedure began by marking the subject with 32 hypoallergenic reflective markers on key anatomical landmarks. The markers were placed on specific anatomical locations based on manufacturer's instructions to help the system analyze the data. Six of the 32 markers (Glabella, Chin, Right and Left Acromion Joint, Right ASIS and Right PSIS) contained special reflective spheres and were placed to make those locations distinguishable in pictures. These six locations play an important role in categorizing the subject's posture and the amount of deviation present.

**Figure 5:**
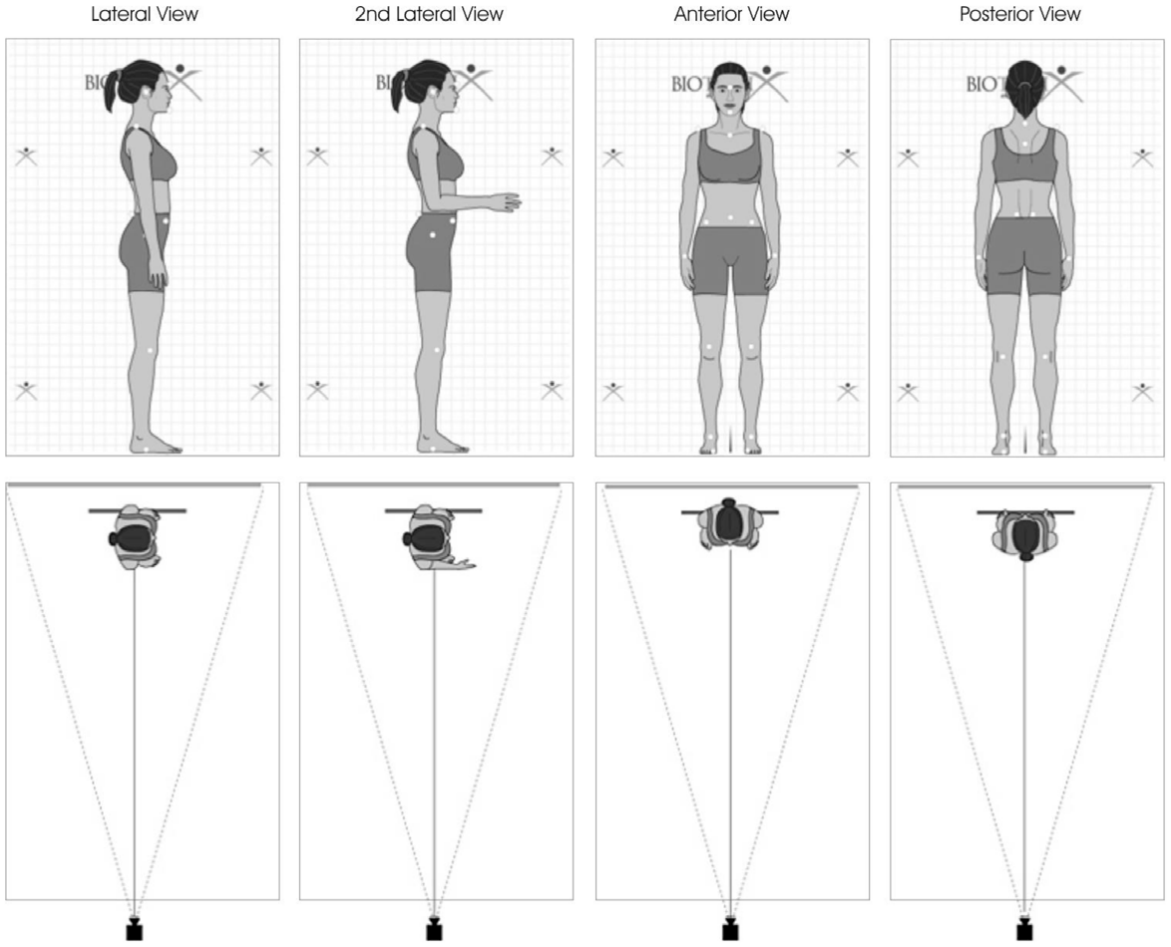
Relative position of subject and digital camera for the Biotonix platform evaluation. Four images were obtained per subject as shown; two lateral views, 1 anterior and one posterior using a digital camera on a tripod at a distance of 9 feet from a calibrated backdrop.

Digital pictures were then taken of the marked subject with a standard digital Kodak DC240 camera (Eastman Kodak Company, Rochester, NY, USA). A set of four photographs were taken per subject (2 lateral views, 1 anterior and 1 posterior) using a digital camera on a tripod at a distance of 9 feet from a calibrated backdrop (**Fig. 5**). For the first photograph, the subject was asked to stand perpendicular to the backdrop, their left foot placed at a marked location on the floor. They were then instructed to take five steps in place, eyes closed, to reset their foot proprioceptors. After this, they were re-centered on the backdrop and asked to inhale and exhale in order to adopt a more natural posture. After achieving this relaxed state, the photograph was taken. For the second photograph, the subject kept the same position, but extended his or her right arm. For the third photograph, the subject stood parallel to the backdrop with both heels on the marker, arms bent at a 90° angle. The subject was then instructed to perform the same actions as on the first photograph to achieve a natural posture. Finally, for the fourth photograph, the subject stood parallel to the backdrop with his or her toes on the floor marker, and was again instructed to take the steps and to inhale-exhale to achieve a natural posture.

### Personality assessment

Subjects were given a Myers-Briggs Type Indicator questionnaire, created by Isabel Briggs-Myers [24]. At the MBTI questionnaire station, they were given instructions on how to properly complete the 93-question test. The room was well lit and quiet, as stipulated in the MBTI protocol.

### Data processing

The digital photographs were subsequently processed through the BioPrint application, which measured and analyzed data from the anatomical markers placed on the subject. The output data consisted of distance and angles of different body segments in three views and two planes, along with the positioning of the body's center of gravity. From this data, the algorithm identified any form of postural deviations, including which the relative strengths of contributing muscles. Analysis included plumb line, angle measurement, compression on different levels of the spine, and center of gravity position.

Subjects were categorized under four different posture types in accordance with Kendall and Kendall's posture categories [11]. The four posture categories are as follows: ideal posture - characterized by neutral position of the head, pelvis and hips, small lordosis of the neck, dorsal kyphosis, and lumbar lordosis; kyphosis-lordosis posture - characterized by forward projection of head, hyperextension of the neck, dorsal hyper-kyphosis, lumbar hyper-lordosis, anterior pelvic tilt, and slight dorsal flexion and hyperextension of the knees; flat back posture - characterized by hyperextension of the hips and knees along with flat lumbar curvature; and sway-back posture - characterized by hyperextension of hips, knees and forward of ankles, and decreased lumbar curvature. For the purpose of this research, only the data from the lateral view were considered in order to comply with Kendall and Kendall's postural observations. All data from the posture analysis, along with data from the pain scale questionnaire, was compiled manually in an Excel document. The MBTI questionnaire was analyzed using the MBTI analysis grid. All MBTI data were also compiled in an Excel document for the final analysis. The postural, pain scale and MBTI data compiled in the Excel document serves as the essential data for this study.

### Statistical analyses

All data are expressed as the mean ± SD and were analyzed using stated student t-test or non-linear regression analysis. Chi-square was used to make decisions about whether a relationship between two or more variables existed. ‘*p*’ value<0.05 were considered significant.

## Supporting Information

Table S1 Results from the Myers-Briggs Personality Type Indicator (MBTI) and postural evaluation of 100 patients involved in the study. The results from the personality inventory (MBTI) came in the form of a combination of letters [Extraversion, Introversion, Sensing, Intuition, Thinking, Feeling, Judging, and Perceiving], and numbers, 1–26. The postural evaluation results came in the form of numbers. The numbers represent data taken from the BioPrint photographs, measuring angles related to each subject's posture. The pain scale data consisted of a number between 0 and 10. After the compilation of all the results in the three categories of testing, one of the four letters [ideal posture (A), kyphosis-lordosis (B), flat back (C), sway-back (D)] was added to the MBTI test results.
(XLS)
